# The effect of an unstructured, moderate to vigorous, before-school physical activity program in elementary school children on academics, behavior, and health

**DOI:** 10.1186/1471-2458-12-300

**Published:** 2012-07-05

**Authors:** Connie L Tompkins, Jacob Hopkins, Lauren Goddard, David W Brock

**Affiliations:** 1 Department of Rehabilitation & Movement Science, University of Vermont, 106 Carrigan Drive, 310D Rowell, Burlington, VT, 05405-0068, USA; 2 Department of Biological Sciences, 528 University of Vermont, 108 Morrill Hall, Burlington, VT, 05405-0068, USA; 3 College of Education and Social Services, University of Vermont, Burlington, VT, USA

**Keywords:** Before-school physical activity, Physical activity and academics, School health

## Abstract

**Background:**

Physical inactivity has been deemed a significant, contributing factor to childhood overweight and obesity. In recent years, many school systems removed recess and/or physical education from their curriculum due to growing pressure to increase academic scores. With the vast majority of children’s time spent in school, alternative strategies to re-introduce physical activity back into schools are necessary. A creative yet underutilized solution to engage children in physical activity may be in before-school programs. The objective of the proposed study is to examine the effect of an unstructured, moderate to vigorous, before-school physical activity program on academic performance, classroom behavior, emotions, and other health related measures.

**Methods/Design:**

Children in 3rd–5th grade will participate in a before-school (7:30–8:15 a.m.), physical activity program for 12 weeks, 3 days a week. Children will be able to choose their preferred activity and asked to sustain physical activity of moderate to vigorous intensity with individual heart rate monitored during each session.

**Discussion:**

The proposed study explores an innovative method of engaging and increasing physical activity in children. The results of this study will provide evidence to support the feasibility of an unstructured, moderate to vigorous, before-school physical activity program in children and provide insight regarding the ideal physical activity intensity and duration necessary to achieve a positive increase in academic performance.

**Trial registration:**

ClinicalTrials.gov Identifier: NCT01505244

## Background

The current guidelines for physical activity recommend that children should partake in regular, moderate to vigorous physical activity for 60 minutes or more each day [1]. Not only are children not meeting the recommended amount of physical activity but schools are also contributing to this culture of physical inactivity. In recent years, many school systems removed recess and/or physical education from their curriculum due to growing pressure to increase academic scores [2]. In addition to the importance of physical activity for overall physical health and fitness, classroom behavior, academic skills, and attention may also improve in children with increasing physical activity [3-5]. There appears to be a positive association between physical activity and academic performance and behavior; however, further research to delineate the ideal duration and intensity is warranted particularly in elementary school children [3-5].

With the absence of physical activity during the school day, after-school programs have become a popular option to help children reach the recommended 60 minutes of activity. Unfortunately, children participating in after-school programs only engage in approximately 20 minutes of moderate to vigorous physical activity with most of their time spent in light or sedentary behavior [5]. Modifying the structure of these school programs in order to better regulate the levels of physical activity in children may seem to be an appropriate response; however, when allowed to engage in unstructured “free-play”, children were significantly more likely to achieve physical activity levels considered to be of moderate to vigorous intensity. Trost et al. [5] observed a 24–55% decrease in moderate to vigorous physical activity during an organized activity period compared to the preferable “free-play”.

A creative yet underutilized potential solution to engage children in physical activity may be before-school programs. Only one study to date was identified which examined the effects of a before-school physical activity program on overall physical activity. Mahar et al. [6] observed children that participated in before-school physical activity were significantly more attentive throughout the day. Furthermore, these children did not become more inactive throughout the day when engaged in early activity [6]. The study by Mahar et al. [6] lends credence to the implementation of a before-school physical activity program to help children achieve the recommended 60 minutes of daily activity without taking time away from academics; however much more research is warranted.

With physical inactivity deemed as a significant contributing factor to childhood overweight and obesity and with the vast majority of children’s time spent in school, this may be the ideal location for implementing physical activity interventions [7]. The objective of the proposed study is to examine the effect of an unstructured, moderate to vigorous, before-school physical activity program on academic performance, classroom behavior, emotions, and other health related measures. The proposed study may provide insight to the relationship between physical activity and academic performance as well as assist in the design of future physical activity interventions. Moreover, to the authors’ knowledge, no study has examined the effect of an unstructured, before-school physical activity program. Therefore, the proposed study protocol represents a novel approach to increasing physical activity in children.

## Methods/Design

### Objectives

This study will aim to implement a 12- week before-school, physical activity program and examine the effect of this unstructured, moderate to vigorous, before-school physical activity program on academic performance, classroom behavior, emotions, and other health related measures. This study will take place at Malletts Bay School in Colchester, Vermont (VT), the 3rd largest community in the state of VT. Approval to conduct the study was received from the Colchester School Board as well as from the principal of Malletts Bay School. Malletts Bay is a public school servicing 3rd, 4th, and 5th graders with a total of 447 students (229 males, 218 females). Approximately 35% of these students are eligible and participate in the Free and Reduced Lunch Program. The proposed protocol was approved by the Institutional Review Board (IRB) of the University of Vermont.

### Participants

Children from Malletts Bay School in grades 3rd, 4th, and 5th grade and ranging in age from 7 to 11 years will be recruited. An IRB approved consent form, group information form, as well as an introduction to the study letter will be sent home with each student. The letter will provide instructions as to how each interested student (along with a parent/legal guardian signature) should complete the consent form and group information form. Students will return their signed consent form along with a completed group information form to their teacher. Study staff will collect and review completed consent forms from the teachers.

Children will indicate on the group information form (included with the consent form) the group (A or B) they would like to participate (Table 1). The investigators will randomize the children to either Group A or Group B on a first-come, first-served basis. Once the maximum capacity for any one group (n = 50) is reached, children will be randomized to the other group if they have indicated their willingness to participate in the other group. Or if the preferred group is at maximum capacity and the child did not request to participate in the other group, then the child would be notified in writing that the requested group was no longer available.

**Table 1:** Group Stratification

**Group A**	Week 0	Physical Activity Program	Week 12
	tests and measures	3 days/week	tests and measures
**Group B**	Week 0	No Physical Activity Program	Week 12
	tests and measures		tests and measures

Once the consent form is received by the study staff, children and parents will be notified through letters mailed to their home address as to what group their child will be participating. The parent(s) of the child will then be contacted by phone to schedule the first testing session.

For those in Group A, an approval from the child’s pediatrician to participate will be requested by the study staff. Parents will provide their child’s medical doctor information on the information form which will be turned in to the child’s teacher along with the signed consent form. Study staff will fax the medical clearance form specific to this study to the child’s doctor. The medical clearance form will include the child’s name, a description of the physical activity program, and a place for the doctor to provide/not provide their medical approval as well as any other medical recommendations. It is mandatory that we have their doctor’s written approval prior to their participation in the physical activity program. Study staff will notify the child’s parent by phone if attempts to obtain medical clearance from the child’s doctor are unsuccessful.

### Intervention

Group A will participate in a before-school (7:30–8:15 a.m.), physical activity program for 12 weeks, 3 days a week (Tuesday, Wednesday, and Thursday). Children will be able to choose their preferred activity (including but not limited to walking, jogging, running, soccer, jump rope, and basketball). Each child will wear a Polar E600 heart rate monitor (chest strap and watch) during each activity session. We will ask each child to engage in moderate to vigorous physical activity with their heart rate ranging from approximately 120 beats per minute to 180 beats per minute. Each child will be provided a target heart rate based on their age before each session. All activity will be freely chosen by study participants while being encouraged to maintain an acceptable heart rate. Heart rate will be recorded and downloaded following each activity session. Study staff will also check and manually record each child’s heart rate a minimum of three times during each 45 minute session.

### Assessments

All participant baseline measures will be performed 1–2 weeks prior to the first physical activity session. Follow-up measures will be performed within 1 week following the final physical activity session. For both Group A and Group B, the testing and measurement sessions will be scheduled at a convenient time before or after school and will be performed in a private room at the school. At the two testing and measurement visits, study staff will obtain weight, height, waist, hip and neck circumferences, resting heart rate and blood pressure for each enrolled child. Children will be asked to complete several questionnaires at both baseline and follow-up regarding their physical activity, eating habits, feelings and emotions, and quality of life. These measurements and questionnaires are expected to take approximately 15–20 minutes. Parent(s) will also be asked to complete questionnaires before and after the physical activity program. The parent questionnaires will include information regarding their education level, their child’s physical activity, eating habits, medications, illnesses, family history and quality of life. These questionnaires will be directly mailed to the parents and will include a postage-paid return envelope. We will also be asking teachers of enrolled participants to complete a brief questionnaire regarding the child’s classroom behavior before and after the physical activity program.

### Measures

#### Anthropometrics

Each study participant’s weight will be measured using an electronically calibrated scale (seca 869 electronic personal scale, seca Ltd, Hamburg, Germany) in kilograms. The participant will wear only one layer of clothing and remove his or her shoes for the weight measure. The study participant will step on the scale, remain as still as possible, and the weight will be recorded as soon as the digital reading is constant. This process will be repeated two additional times and the average weight in kilograms of the 3 measures will be calculated and recorded on the data statistical form. Height will be measured without shoes using a transportable stadiometer (seca 213 Stadiometer, seca Ltd, Birmingham, UK) to the nearest 0.1 cm. Study participants will be asked to stand upright with their heels against the board while facing the study personnel performing the measurement. Height will be recorded on the data form. Using the participants’ height and weight, body mass index (BMI) will be calculated and recorded.

Waist, hip, and neck circumference will also be measured. Waist circumference will be measured at the level of the umbilicus to the nearest 0.1 cm. Hip circumference will be measured at the level of the greatest posterior protuberance of the buttocks. Neck circumference will be measured in the midway of the neck, between midcervical spine and midanterior neck, to within 1.0 mm. All circumference measures will be taken with the study participant standing upright and facing the study personnel performing the measurement.

#### Physiological measurements

Blood pressure and resting heart rate will be assessed with the BP760 Omron Blood Pressure Monitor with ComFit Cuff. The study participant will sit quietly for at least five minutes prior to the measurements to allow heart rate to reach a rested baseline. Two readings of both blood pressure and heart rate will be obtained and averaged. For blood pressure, if the two values differ by more than 5 mmHg, an additional reading will be obtained. A small cuff will be used for the children. The study participants will be instructed to abstain from heavy exercise for at least two hours prior their heart rate and blood pressure measures. Study participants will also be instructed not to ingest caffeine within 30 minutes before their resting heart rate and blood pressure measurements. The study participant will be comfortably seated, with the arm slightly flexed, palm up and the entire forearm supported at heart level on a smooth surface.

#### Academic performance

Academic performance will be assessed through several methods including grades, achievement test scores, and curriculum-based measures (progress monitoring). The curriculum-based measures are a unique method as these are sensitive to and are validated to monitor progress compared to curriculum-based assessments. Curriculum-based assessments are often lengthy, not administered on a regular basis, and compare student scores to national samples. Curriculum-based measures are easy to administer, can be given as often as wanted/needed, and provide immediate feedback. Progress monitoring with curriculum-based measures focuses on individualized decision making in general and special education with respect to academic skill development at the elementary grades. Progress monitoring can be conducted frequently and is designed to estimate rates of improvement [8].

The proposed study will be utilizing two curriculum based measures developed by Pearson based on the work by Fuchs and Fuchs. The first is the M-COMP, which measures progress in basic number operations (addition, subtraction, multiplication, division) through grade 8. The second measure is the Oral Fluency Measure which measures progress in comprehension, reading, vocabulary, phonics etc. Criterion and norm based reference points are available. The norms are established through an aggregate score of the students in U.S. using the Pearson measures [8]. The curriculum-based measures will be performed 1–2 weeks prior to the first physical activity session. Follow-up measures will be performed within 1 week following the final physical activity session.

### Questionnaires

#### Physical activity

Participants’ physical activity will be assessed with the Physical Activity Questionnaire for Older Children (PAQ-C) administered by the study staff [9,10]. The PAQ-C was developed and validated to compare and measure moderate to vigorous physical activity levels over a 7-day period among elementary school aged children.

#### Eating behavior

The University of California Cooperative Extension Food Behavior Checklist [11] will be administered to assess participant’s eating behavior. This validated 16-item food behavior checklist has an elementary reading level and includes six subscales -milk, fat/cholesterol, diet quality, food security, and fruit/vegetable.

#### Feelings and emotions

Feelings and emotions will be measured with the Positive and Negative Affect Scale, Child Version (PANAS-C) [12]. The PANAS-C was developed mainly as an instrument for use in a general school population as a brief measure of emotional experience that can be used to differentiate anxiety from depression in children. The PANAS-C is very simple (27 items) as children will be instructed to indicate how often they have felt a specific way (e.g., interested, sad etc.,) during the “past few weeks” with a 5-point Likert scale (1 = very slightly or not at all, 5 = extremely).

#### Health-related quality of life

Health-Related Quality of Life (HRQOL) will be assessed with the Pediatric Quality of Life Inventory (PedsQL) [13]. The PedsQL is a 23-item, reliable and valid tool for measuring HRQOL in children. The PedsQL is comprised of four multi-dimensional scales that include physical, emotional, social and school functioning which produce three summary scores of psychosocial health, physical health and a total scale score to measure HRQOL. The PedsQL instruments are sensitive to cognitive development and consist of developmentally appropriate forms along with a parent proxy-report for children ages 8–12 years [13].

#### Data analysis

All statistical procedures will be performed in SPSS, version 18.0. Significance will be set at the 0.05 level. Descriptive statistics including means and standard deviations will be computed for all of the data. Differences among the groups at Week 0 and Week 12 will be assessed by using Student’s independent *t*-test. Furthermore to account for baseline differences, a general linear mixed-model analysis of variance will be performed. A power analysis was performed to determine the sample size needed to determine significant pre- and post-differences in each group. With the alpha level set at 0.05, an anticipated effect size of 0.8, and the statistical power level set at 0.8, the minimum total required sample size was 8 participants. A power analysis was performed to determine the sample size needed to determine significant differences between Group A and Group B with a one-tailed *t*-test. With the alpha level set at 0.05, an anticipated effect size of 0.8, and the statistical power level set at 0.8, the minimum total required sample size was 42, with a minimum sample size per group of 21 participants. Therefore, with the planned total of 100 participants in this study, 50 of which will be assigned to Group A and 50 assigned to Group B, the minimum sample size required for analyses will be achieved.

## Discussion

The proposed study described in this paper is the first to the authors’ knowledge to examine the effect of an unstructured, moderate to vigorous, before-school physical activity program on academic performance, classroom behavior, emotions, and other health related measures in elementary school-aged children. The results of this study will provide evidence to support the feasibility and efficacy of an unstructured, moderate to vigorous, before-school physical activity program in children. The unstructured physical activity with limited equipment, yet moderate to vigorous intensity design would allow others to easily and affordably replicate. Often, the initiation of novel physical activity programs is often viewed as cost-prohibitive and labor intensive. The design, setting, and population, however, may be replicated in relatively any elementary school with limited supplies and with a limited budget. Furthermore, the proposed study will provide insight regarding the ideal physical activity intensity and duration necessary to achieve a positive increase in academic performance.

The current study allows the participants to select and/or indicate their preferred group- Group A, tests and measures with the physical activity intervention or Group B, tests and measures only. This randomization design may be considered a limitation of the study; however, differences within each group, treatment and control will be assessed in addition to comparisons between the two groups. These within-group analyses will allow evaluation of the outcomes attributable to the physical activity intervention rather than attitudinal variables that may have been introduced with the self-selection of the groups.

The findings of this study will provide valuable information for other research groups looking to increase physical activity levels of children via school-based interventions. Furthermore, it will ascertain whether an unstructured, moderate to vigorous, before-school physical activity program is an effective method for future large-scale implementation.

## Competing interests

The authors declare that they have no competing interests.

## Authors’ contributions

CT and DB conceived of the study, participated in its design, and contributed to developing the protocol. All authors helped to draft and review the manuscript and CT edited and approved the final version of the paper. All authors read and approved the final manuscript.

## Pre-publication history

The pre-publication history for this paper can be accessed here:

http://www.biomedcentral.com/1471-2458/12/300/prepub
